# The Application of the NGS and MLPA Methods in the Molecular Diagnostics of Lynch Syndrome

**DOI:** 10.3390/diagnostics15232950

**Published:** 2025-11-21

**Authors:** Ivana Rako, Ema Vinceljak, Marina Popovic, Tamara Zigman

**Affiliations:** 1 Clinical Department of Laboratory Diagnostics, University Hospital Center Zagreb, 10000 Zagreb, Croatia; 2 Faculty of Pharmacy and Biochemistry, University of Zagreb, 10000 Zagreb, Croatia; 3 Department of Oncology, University Hospital Center Zagreb, 10000 Zagreb, Croatia; marina.baric11@gmail.com; 4 School of Medicine, University of Zagreb, 10000 Zagreb, Croatia; tzigman1@kbc-zagreb.hr; 5 Department of Pediatrics, University Hospital Center Zagreb, 10000 Zagreb, Croatia

**Keywords:** Lynch syndrome, MMR genes, NGS, MLPA

## Abstract

**Background**: Lynch syndrome (LS) is a cancer-susceptibility syndrome associated with autosomal dominant predisposition to a spectrum of cancers, primarily of the colorectum and endometrium, which exhibit impaired DNA mismatch repair (MMR) activity. LS is caused by a hereditary (germline) pathogenic (PV) or likely pathogenic variant (LPV) in one of the mismatch repair (MMR) genes—*MLH1*, *MSH2*, *MSH6*, *PMS2*, or *EPCAM*. Although point mutations are the most common genetic changes in MMR genes, >20% are large genomic rearrangements. We hypothesized that a two-tier diagnostic strategy for Lynch syndrome (LS) using next generation sequencing (NGS) and multiplex ligation-dependent probe amplification (MLPA) can increase diagnostic yield of patients with Lynch syndrome. **Methods**: This study included 60 patients suspected of LS. After genetic counseling, they were referred to genetic testing. Genomic DNA was extracted from peripheral blood and sequenced using NGS multigene panel testing covering 113 cancer susceptibility genes, including MMR genes. Regarding limitations of NGS analysis, which cannot reliably detect genomic alterations larger than 50 base pairs in length, the MLPA method was used for NGS negative DNA samples in order to identify larger deletions and duplications, commonly referred to as copy number variations (CNVs). **Results**: Different PVs were detected by NGS in 10 patients and CNVs were detected by MLPA in 7 more patients: 3xMLH1 del ex9-15, 2xMSH2 del ex1 and upstream, 1xMSH2 del ex9, and 1xMSH2 del ex1. We did not detect LPVs or variants of uncertain significance (VUS). In our cohort, the addition of MLPA provided an incremental yield of seven pathogenic CNVs, representing an 11.6% absolute increase in diagnostic sensitivity (from 16.7% to 28.3%) over the NGS-alone workflow, with CNVs accounting for 41% of all pathogenic findings. **Conclusions**: Our results show that MLPA is a very useful method in molecular diagnostics of LS and its implementation in routine genetic testing in combination with NGS using multigene panel testing would benefit both patients and health care providers.

## 1. Introduction

Lynch syndrome (LS) is one of the most common hereditary syndromes associated with colorectal cancer (CRC), accounting for 3–4% of all hereditary cancer syndromes [1]. Individuals with Lynch syndrome not only have an increased risk of developing hereditary nonpolyposis colorectal cancer (HNPCC) but also have a significantly higher risk for a wide spectrum of cancers beyond the colon such as endometrial, ovarian, breast, and urothelial cancers.

Individuals with Lynch syndrome carry a heterozygous hereditary pathogenic or likely pathogenic variant (PV or LPV) in one of the tumor suppressor genes involved in mismatch repair (MMR genes), *MLH1, MSH2, MSH6, PMS2*, or *EPCAM*, which leads to the loss of one functional copy of these genes [2]. This condition is inherited in an autosomal dominant manner, meaning that inheriting just one mutated allele is sufficient to increase cancer risk.

Around 90% of LS cases are caused by *MLH1* and *MSH2* gene mutations, while about 10% of LS patients carry *MSH6* and *PMS2* mutations [3]. Mutations in the *EPCAM* gene are extremely rare, occurring in only about 3% of cases [2].

The mismatch repair (MMR) system is essential for maintaining genomic stability and integrity in the cell [4]. Patients with LS have a heterozygous hereditary mutation in one of the MMR genes. However, to initiate tumorigenesis, both copies of the gene must be dysfunctional in the tumor cells, meaning a second genetic alteration must occur in the remaining healthy allele, leading to loss of heterozygosity (typically through somatic, acquired changes), resulting in complete loss of MMR protein expression in the tumor.

When MMR is nonfunctional in a cell, accumulation of mutations, particularly in microsatellite regions of DNA, occurs. The accumulation of mismatched bases in microsatellite regions phenotypically results in microsatellite instability (MSI) [1,2]. In MMR-deficient tumor cells, the length of microsatellite nucleotide repeats differs from that of the repeats in surrounding healthy tissue [5]. Therefore, MSI is one of the main molecular characteristics of nonpolyposis CRC within Lynch syndrome. Since over 90% of CRC associated with LS is described as microsatellite-unstable, MSI can be considered a reliable marker of MMR deficiency [5]. This characteristic is indicated in histopathological findings as MSI-H (high microsatellite instability).

The diagnostics of LS begin with genetic counseling, where individuals at increased risk for cancer are identified based on personal or family history and other characteristic features of hereditary cancer syndromes. Accordingly, they are referred to genetic testing, regular preventive screenings, or preventive pharmacological and/or surgical interventions. Early diagnosis increases the survival rate of affected individuals, as well as probands relatives who undergo testing and surveillance [2]. Therefore, before and after genetic testing, genetic counseling must occur.

The majority of changes in MMR genes are point mutations, but there are also splice-site mutations and frameshift mutations followed by large genomic rearrangments. Large genomic rearrangements, usually defined as alterations encompassing at least 50 base pairs in length, are called structural variants. Structural variants mostly include large deletions and duplications/insertions, which can produce genomic imbalances called copy number variants (CNVs) [6]. Due to the presence of different genomic changes in MMR genes, the diagnosis of LS requires a combination of molecular methods that can detect all possible alterations in these genes. This comprehensive approach is critical for accurate diagnosis and effective management of individuals at risk for Lynch syndrome-related cancers.

Compared to traditional molecular methods, NGS is faster, broader, and more cost-effective. It can be used for singlegenes, targeted multi-gene panels, whole exomes, or even whole genomes, giving complete genetic information but keeping costs controllable. In oncology, NGS multi-gene panel testing (MGPT) is favored due to its higher analytical sensitivity and specificity. By focusing on cancer-associated genes, it simplifies interpretation and improves diagnostic accuracy. NGS uses massive parallel sequencing of DNA fragments to detect genetic changes by analyzing read depth, while MLPA is a semi-quantitative technique based on the amplification of probes, each detecting a specific DNA sequence. It can detect deletions or duplications as small as 60 nucleotides, which allows for the detection of single exon deletions or insertions/duplications. NGS provides high throughput and the ability to analyze large gene panels in a single experiment, while MLPA is a more targeted, cost-effective, and labor-intensive method used for specific genes or regions, often as a confirmatory test for NGS results.

In this study, we hypothesized that combination of two complementary molecular methods could increase diagnostic sensitivity in molecular diagnostics of LS: next-generation sequencing multigene panel testing for detection of point mutations, frameshift mutations, splice-site mutations, small deletions, and small duplications/insertions and multiplex ligation-dependent probe amplification (MLPA) for detection of deletions and duplications/insertions (CNVs) larger than 50 base pairs in length.

## 2. Materials and Methods

In University Hospital Center Zagreb, after genetic counseling, 60 patients suspected of Lynch syndrome were referred to the Department of Molecular Laboratory Diagnostics for genetic testing from 2022 to 2024. Our cohort included 60 participants; 52 participants had a personal history of cancer and 8 participants were included due to positive family history suggestive of Lynch syndrome. Average age was 51.2 years (±12.8 SD, 29–78). There were 16 males and 46 females. The youngest patient confirmed to have Lynch syndrome was a 30-year-old female patient affected by colorectal cancer. A total of 24 participants had colorectal cancer; 12 of them had endometrial carcinoma and 5 of them had urothelial carcinoma.

As inclusion criteria, we used Amsterdam II clinical criteria as follows:

There should be at least three relatives with HNPCC-associated cancer (cancer of the colorectum, endometrium, small bowel, ureter, or renal pelvis) and the following:

- One should be a first-degree relative to the other two;
- At least two successive generations should be affected;
- At least one should be diagnosed before age 50;
- Familial adenomatous polyposis should be excluded;
- Tumors should be verified by pathological examination.

To identify patients eligible for molecular testing for MSI, revised Bethesda criteria were used as follows:

- Individual with CRC diagnosed by age 50;
- Individual with synchronous or metachronous CRC, or other HNPCC-associated tumors regardless of age;
- Individual with CRC and MSI-H histology diagnosed by age 60;
- Individual with CRC and more than 1 first-degree relative (FDR) with an HNPCC-associated tumor, with one cancer diagnosed by age 50;
- Individual with CRC and more than 2 FDRs or second-degree relatives (SDRs) with an HNPCC-associated tumor, regardless of age.

This study was conducted in accordance with the Declaration of Helsinki and approved by the Ethics Committee of University Hospital Centre Zagreb for studies involving humans (Class 8.1-24/257-2, No 02/013AG, 4 December 2024). An informed consent statement was obtained from the participants included in the study. After peripheral blood sampling (K3EDTA, 3 mL tubes), genomic DNA extraction was performed (QIAamp DNA Blood Mini Kit, Qiagen, Germany). In all DNA samples obtained by extraction, DNA concentration and purity were checked by spectrophotometric and fluorometric measurement (NanoDrop and Qubit). For all patients, DNA samples were sequenced using next-generation sequencing (NGS) with a multigene panel covering 113 cancer susceptibility genes, including MMR genes (TruSight hereditary cancer panel, MiSeq, Illumina). The analysis was performed using the software package provided by the manufacturer (Variant Studio software package, Illumina). The NGS multigene panel testing covering 113 cancer susceptibility genes including MMR genes used predesigned, ready-to-use oligo probes that covered exonic regions for all genes and 20 bp of flanking intronic regions for each targeted gene including MMR genes (*MLH1*, *MSH2*, *MSH6*, *PMS2*, and *EPCAM*). For secondary and tertiary NGS data analysis, we used DRAGEN v4.3 and EMEDGENE v38 software with set criteria for germline variants: read depth threshold of 50x and variant frequency (VAF) ≥ 20%. For our NGS multi-gene panel, the mean read depth for targeted sequences ranged from about 300 to 600x between runs for different regions, including MMR genes. After the validation process, using Sanger sequencing for PV, LPV, and VUS confirmation, we determined that the NGS software package we used had a limitation in genomic variant detection at an upper limit of 50 base pairs in length. Therefore, DNA samples with no pathogenic variant in MMR genes detected by the NGS were further analyzed by the MLPA method for the presence of copy number variants. The reagents used for the MLPA analysis were provided by MRC Holland (Amsterdam, The Netherlands)—Probemix P003 (IVD) MLH1/MSH2, Probemix P008 (IVD) PMS2, Probemix P072 (IVD) MSH6-MUTYH, and Probemix P248 (IVD) MLH1-MSH2 Confirmation. In order to perform *EPCAM* gene testing, we used SALSA MLPA Probemix P072 MSH6-MUTYH (IVD), which detected CNVs in the *MSH6* gene, the *EPCAM*/*MSH2* region, and the *MUTYH* gene. For the *EPCAM* gene, the MLPA probes were arranged to cover exons 3, 8, and 9 and a downstream region (3′-end of EPCAM). The NGS method provided good coverage for all exons in the PMS2 gene (300–600x) by using NGS multi-gene panel testing where *PMS2*-*PMS2CL* homology (pseudogene) was solved by a novel computational method called multi-region joint detection (MRJD). Instead of considering each region in isolation and genotyping them individually, MRJD considers all locations from which a group of reads may have originated and attempts to detect the underlying sequences jointly. This approach retains reads with ambiguous alignment and is useful for instances of read misalignment due to gene conversion or crossover events. The SALSA MLPA Probemix P008 PMS2 is an in vitro diagnostic (IVD) semi-quantitative assay for the detection of deletions or duplications in exons 1–11 of the PMS2 gene and in exons 12–15 of the *PMS2* gene or *PMS2CL* pseudogenes. Since we did not find a VUS/LP/P variant in the *PMS2* gene, it was not necessary to confirm it using confirmation methods. For fragment analysis, a capillary electrophoresis device was used (ABI 3500xL Genetic Analyser, Applied Biosystems, Waltham, MA, USA). The results were analyzed using the Coffalyser.Net software tool provided by MRC Holland.

For variant interpretation, various databases were used (ExAC, gnomAD, 1000 Genomes, ClinVar, OMIM, COSMIC, PubMed, etc.). In silico programs (SIFT, PolyPhen, and Mutation Tester) were used to assess the pathogenicity of the variants. Sequence changes were defined based on the reference genome (GRCh37) and interpreted in the context of the clinically relevant transcript (NM_). All detected pathogenic or likely pathogenic variants were classified and reported according to ACMG standards and guidelines for the interpretation of sequence variants [7].

Tumor-based analysis of Mismatch Repair (MMR) status was performed by accredited pathology services, including the Clinical Hospital Centre Zagreb (KBC Zagreb), following established national and international guidelines for Lynch syndrome screening. For immunohistochemistry (IHC), a four-antibody panel (MLH1, PMS2, MSH2, and MSH6) was used. The universal standard of loss of nuclear staining in tumor cells (with retained positivity in internal control cells) was utilized as the cut-off to define protein deficiency. For microsatellite instability status (MSI), testing was conducted using either the five-marker NCI panel (BAT25, BAT26, D5S346, D2S123, and D17S250) or an equivalent, validated commercial multiplex system. In line with consensus guidelines, MSI-High (MSI-H) was defined as instability in ≥2 of the 5 markers.

## 3. Results

Our study included 60 patients, 14 male (median age 49 (IQR: 40–54)) and 46 female (median age 51 (IQR: 41–63)). Almost every selected patient had previously developed some type of cancer associated with LS (colorectal, endometrial, ovarian). Only 8 out of 60 patients had not previously developed cancer but were suspected of LS regarding positive family history. Out of 60 patients, a total of 10 genetic changes in MMR genes were detected by NGS method, which corresponds to 16.7% of patients (10/60). For the patients with pathogenic variants in MMR genes detected by NGS, there was no need to perform MLPA analysis. Therefore, MLPA analysis was performed on 50 patients, where seven additional copy number variants were detected with the MLPA method, representing 14% of NGS negative patients (7/50). By using two complementary methods, a total of 17 pathogenic variants in MMR genes were detected, corresponding to 28.4% of total patients (17/60). No LPs or VUSs were detected. Of all detected variants, point mutations, frameshift mutations, and splice-site mutations account for 59% (10/17) and CNVs account for 41% (7/17). All PVs detected by NGS were confirmed with Sanger sequencing, while CNVs detected in *MLH1* and *MSH2* genes by MLPA were confirmed using Probemix P248 (IVD) MLH1-MSH2 Confirmation, as recommended by the manufacturer.

Data relevant to LS diagnosis for the patients with MMR pathogenic variants are presented in Table 1.

**Figure 1:**
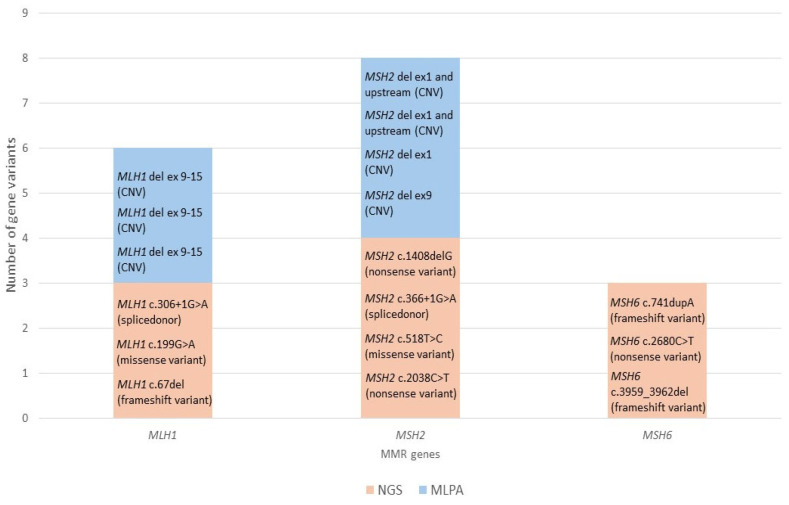
Genetic changes in MMR genes expressed by specific gene and method. Ten PVs were detected in the *MLH1*, *MSH2,* and *MSH6* genes by NGS (orange): three in the *MLH1* gene, four in the *MSH2* gene and three in the *MSH6* gene. Seven PVs were detected in the *MLH1* and *MSH2* genes by MLPA (blue): three in the *MLH1* gene and four in the *MSH2* gene. No variants in the *PMS2* and *EPCAM* genes were detected.

The majority of changes were detected in the *MSH2* gene (8/17; 47.1%), followed by the *MLH1* gene (6/17; 35.3%), and 3 out of 17 were detected in *MSH6* (3/17; 17.7%). Figure 1 graphically shows genetic changes found in our patient group. No variants in the *PMS2* and *EPCAM* genes were detected. Interestingly, all PVs detected by NGS were different. There were three nonsense, three frameshift, two missense, and two splice donor pathogenic variants, whereas the CNVs detected by MLPA were repeated: all three changes detected by MLPA in the *MLH1* gene were the same deletions of exons 9–15 and two of the four changes detected by MLPA in the *MSH2* gene were the same deletions of exon 1 and the upstream region. In the *MSH6* gene CNVs were not detected.

Microsatellite instability (MSI) and/or immunohistochemical staining (IHC) were available for 13 patients where, in 11 tumor tissue samples, MSI and/or positive IHC results (lack of MMR protein expression) were confirmed, but in two tumor tissue samples, MSI was negative (sustained MMR protein expression). In five patients with positive hereditary PVs in MMR genes, the MSI and/or IHC testing results in tumor tissue for the same gene were also positive (lack of certain protein expression in tumor tissue corresponds to the mutation in the gene). However, six patients had tumors with MSI and/or positive IHC results but no detected hereditary PV in their MMR genes. Table 2 shows the concordance of MSI/IHC-positive results with the germline findings per case.

**Figure 2:**
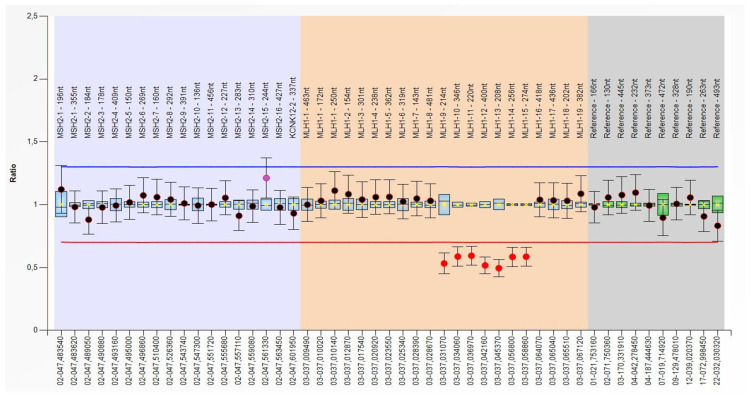
Molecular diagnosis of a large genomic rearrangement in the *MLH1* gene by multiplex ligation-dependent probe amplification (MLPA). An example of a positive MLPA result (*MLH1* del ex 9–15)**.** The result of analysis is shown as a graphical representation of normalized ratio analysis (MLPA-probe ratios in the patient sample versus healthy donor control demarked as reference sample). The Y-axis represents the Normalized Probe Ratio (Signal Sample/Signal Reference). The expected ratio for two normal gene copies is 1.0 (indicated by the central horizontal line, black spots). Probes falling below the deletion cut-off (0.70 to 0.5) indicate a gene deletion (red spots). The X-axis represents probe locations based on hg18/mapview build 36. Mapview location consists of the chromosome no. + distance (nt) from the p-telomere to the start of the probe sequence. This specific result demonstrates a partial germline deletion of *MLH1* gene spanning exons 9 to 15. Probes for these exons show a clear signal reduction, resulting in a ratio of approximately 0.50 (red spots, indicating a single-copy loss), while the other exon probes and all internal reference probes remain within the normal range (approximately 1.0).

Figure 2 shows positive MLPA results for a large heterozygous deletion that includes seven exons (exon 9–15) in the *MLH1* gene. For the same patient (Sample number 4), a lack of MLH1 protein expression was demonstrated in their tumor tissue (colon cancer).

## 4. Discussion

In Lynch syndrome, the risk for tumor development is inherited, rather than the disease itself [8]. The risk of developing CRC varies depending on gender and the affected MMR gene [1]. In addition to CRC, there is also a significantly increased risk for the development of other cancers outside the colon, again depending on the affected MMR gene and gender [1]. Endometrial cancer (EC) is the most common cancer outside the colon associated with LS, occurring in women at a rate comparable to that of colorectal cancer. The risk of developing EC ranges from 14 to 54% when the *MLH1* gene is mutated, 17–71% when the *MSH6* gene is mutated, and 15% when the *PMS2* gene is mutated. The risk for ovarian cancer is lower, at 4–20%, and is mainly associated with mutations in the *MSH2* gene.

In our cohort of 60 patients, LS was confirmed in 17 patients who had germline PV in one of the MMR genes. In genetic counseling, 15 of the 17 patients already had a diagnosis of cancer, mostly colon cancer (11/17) and endometrial cancer (4/17), but also breast, urothelial, ovarian, gastric, and cervical cancer, where five patients had two or three different primary cancers necessarily including colon or endometrial cancer. Two patients with detected PVs in MMR genes were healthy but with extremely positive family histories of LS cancers (see Table 1).

According to the literature, in approximately 80–90% of LS cases, the mutations affect the *MLH1* and *MSH2* genes, while in the remaining 10–20% of LS cases, mutations involve the *MSH6* or *PMS2* genes. Mutations in the *EPCAM* gene are extremely rare, occurring in only about 3% of cases [2]. A 3′ deletion of the terminal exons of the *EPCAM* gene, which is located upstream of *MSH2* gene, causes epigenetic silencing of *MSH2*, resulting in a tissue-specific deficiency of the MSH2 protein [9]. The most common point mutations in MMR genes are missense mutations which lead to single amino acid substitutions. Additionally, there are nonsense mutations, where the substitution of a single nucleotide introduces a stop codon, leading to truncation of the polypeptide chain, again resulting in a nonfunctional protein [10] (pp. 63–67). The frequency of missense mutations for all four genes (*MLH1, MSH2, MSH6, PMS2*) ranges from 30% (*MSH2*) to 60% (*PMS2*). Also, common pathogenic variants in *MLH1*, *MSH2*, and *MSH6* are truncating variants (predominantly nonsense or frameshift mutations) with a frequency from 40% (*MLH1*) to 49% (*MSH2*), while splice-site pathogenic variants occur least frequently, from 3% (*MSH6*) to 11% (*MLH1*) [11]. A significant proportion of changes in MMR genes are copy number variants or large genomic rearrangements. According to Cohen et al. (2019), large deletions account for 22% of *MLH1* and *PMS2* mutations, 26% of *MSH2* mutations, and about 7% of *MSH6* mutations [12]. In the *EPCAM* gene, large genomic changes are the only alterations reported in the literature to date [13].

In this study, the highest number of PVs were found in the *MSH2* gene (8/17; 47.1%) of which four large deletions were detected by the MLPA method and four PVs detected by the NGS method: two nonsense, one missense, and one splice donor. In the *MLH1* gene, six variants were detected (6/17; 35.3%), including three large genomic deletions, while the remaining three PVs were detected by the NGS method: one frameshift, one missense, and one splice donor. Additionally, in the *MSH6* gene, three PVs were detected by NGS (3/17, 17.6%), two frameshift and one nonsense, while no genomic changes were detected in the *PMS2* and *EPCAM* genes (see Figure 1).

To detect all possible molecular changes in MMR genes, it is advisable to use a method that covers as many genes as possible. The most commonly used method is NGS multigene panel testing, which can detect point mutations, as well as small deletions and insertions/duplications in several hundred genes simultaneously [2]. However, depending on the software package used for analyzing NGS data and known software limitations, this method cannot reliably detect SVs, such as large deletions and/or insertions/duplications, structural rearrangements, or genetic changes in gene regions not covered by the multigene panel applied. Therefore, supplementary methods should be used to detect the presence of SVs as these changes account for a significant portion (>20%) of all changes in MMR genes. In our sample, out of seventeen detected variants, three were nonsense, three frameshift, two missense, two splice donor variants, and seven CNVs. As a first molecular method, we used NGS multigene panel testing (NGS) and, as a second method, we used MLPA, which can detect SVs such as exon-level CNVs. With such a combination of methods, we detected 17 pathogenic variants, where 10 variants were detected by NGS, and 7 were large deletions from 1 to 6 exons in size, detected by MLPA.

Our results are similar to data in the literature. We performed genetic testing on a group of 60 patients. Pathogenic or likely pathogenic variants were identified in the *MLH1* and *MSH2* genes of 21 patients (21/60, 35%), and approximately one-third of these changes were large deletions. In our study, 17 pathogenic changes were detected, which corresponds to 28.3% (17/60), and is in line with the results of other studies available in the literature [14]. Gylling et al. (2009) [15] examined changes in MMR genes in 45 patients who did not have point mutations identified by sequencing. They found a large genomic change in 12 out of 45 patients (27%): deletions were present in 3/25 (12%), 9/16 (56%), and 0/4 (0%) of patients with missing expression of MLH1, MSH2, and PMS2 proteins, respectively [15].

The clinical criteria for the initial identification of LS cases are summed up by the Amsterdam criteria and the revised Bethesda guidelines [2]. In 1990, the International Collaborative Group on Hereditary Non-Polyposis Colorectal Cancer (HNPCC) developed the Amsterdam Criteria I for identifying families that could be candidates for genetic testing. These criteria were later expanded to include cancers outside the colon that occur as part of LS (Amsterdam Criteria II). However, these criteria proved to be very restrictive, resulting in missed identification of some carriers of mutations in MMR genes [5]. The revised Bethesda guidelines for microsatellite instability (MSI) testing provide detailed instructions on when to test a patient for MSI [2]. Nevertheless, the Amsterdam criteria (I and II) and the revised Bethesda guidelines are not always sufficient for identifying LS patients, which is why they are not fully implemented in clinical practice [9]. When a patient meets the Amsterdam criteria or at least one of the revised Bethesda guidelines, testing for microsatellite instability (MSI) and/or immunohistochemical staining (IHC) is recommended if tumor tissue is available [2]. However, today, a more widely accepted approach is universal IHC/MSI testing for all newly diagnosed colorectal cancers, regardless of whether the guidelines are met, as it has been observed that strict adherence to the guidelines can lead to misdiagnosis of LS [1]. In patients with MSI-high (MSI-H) tumors or loss of MMR protein expression in tumor tissue, further genetic testing is performed to identify the mutation or change in the specific gene that caused MSI or loss of MMR protein expression [2]. Genetic testing can detect mutations in tumor tissue or peripheral blood. Since confirmation of the LS diagnosis is based on identifying the hereditary mutation, the preferred sample is peripheral blood, which is followed by targeted testing of family members of the proband with the purpose of disease prevention [2]. Robinson et al. (2007) [16] investigated the presence of hereditary mutations in MMR genes in a group of 112 patients. Patients were selected based on the Amsterdam criteria, the presence of microsatellite instability (MSI) in the tumor, and the results of immunohistochemical analysis. They identified a hereditary mutation in 69 out of 112 patients. They found pathogenic hereditary mutations in MMR genes not only in patients with MSI-H tumors but also in patients with MSI-negative tumors. This demonstrated that hereditary mutations in MMR genes can be found in patients with MSI-negative tumors, and also that patients with MSI-H colorectal cancer (CRC) or loss of MMR protein expression in their tumor may not necessarily show hereditary mutations in MMR genes through genetic testing, which would exclude the diagnosis of Lynch syndrome [16]. Out of the 60 patients recruited in our study, MSI and/or IHC testing results were available for 13 patients. For 11 of them, MSI and/or positive IHC testing results (lack of MMR protein expression) were confirmed in tumor tissue, but in 2 patients, MSI was negative (sustained MMR protein expression) in tumor tissue. Furthermore, out of 60 patients, 6 patients had tumors with MSI and/or positive IHC results but no detected pathogenic variants in MMR genes. Out of 17 patients with a confirmed pathogenic variant in one of the MMR genes, 5 patients had available MSI and/or IHC testing results in tumor tissue (see Table 1), and in those 5 cases, MSI and/or IHC testing results perfectly correlated with NGS and/or MLPA results (lack of certain protein expression in tumor tissue corresponds to the mutation in the gene). The six patients displaying a deficient MMR (dMMR) tumor phenotype (positive MSI/IHC findings) without a detectable germline pathogenic variant (PV) represent the clinical challenge of ‘Lynch-like Syndrome.’ These findings warrant extended genetic investigation for alternative causes, most notably acquired somatic hypermethylation (epimutation) of the MLH1 promoter in cases of MLH1/PMS2 loss, or the presence of PVs in less common MMR genes or in intronic/deep regulatory regions not covered by current molecular methods.

The MLPA method for MMR gene testing has an IVD (In Vitro Diagnostic) certificate that demonstrates that a medical device used to test samples taken from the human body (like blood or tissue) complies with the requirements of the European Union’s In Vitro Diagnostic Regulation (IVDR). It signifies that the device is safe and performs as intended for its intended use, allowing it to be legally commercialized within the EU, establishing a quality management system (QMS), preparing technical documentation, and ensuring that the device undergoes conformity assessment. However, the MLPA method has certain limitations that need to be considered before its implementation in routine diagnostics. For example, the presence of a single nucleotide polymorphism (SNP) or point mutation at the probe binding site can prevent the probe from binding and amplifying, which could then be interpreted as a false-positive deletion. Further, MLPA method cannot detect balanced gene rearrangements and mutations in parts of the genome that are not covered by the probes, so a negative result does not exclude translocation or inversions or the presence of a genomic change at a genomic position not covered by the probes. The greatest advantage of the MLPA method is the ability to simultaneously test whole genes and a large number of samples, which facilitates routine application. Additionally, the test manufacturer provides users with appropriate software for processing data obtained through capillary electrophoresis. However, in the future, the MLPA method could be replaced by long read sequencing technology, which generates long, continuous DNA sequences (kilobases to megabases), making it ideal for resolving complex structural variations and repetitive regions, whereas MLPA is a more targeted and sensitive method for detecting specific, known CNVs like deletions or duplications at specific locations in a gene.

## 5. Conclusions

In our cohort of 60 well selected patients suspected of LS after genetic counseling, we were able to demonstrate the importance of combining different molecular methods in order to increase diagnostic sensitivity in confirming the diagnosis of LS. Since our results showed that hereditary PVs in the MMR genes were detected by NGS method in 16.7% patients, and by a combination of NGS and MLPA methods in 28.3%, we concluded that the use of the MLPA method as a complement to NGS multigene panel testing increased the identification individuals with LS.

Although the most frequently hereditary PVs in MMR genes can be detected using NGS multigene panel testing, large deletions and insertions/duplications require additional CNV analysis. When the bioinformatics pipeline lacks a solution for CNV analysis from NGS multigene panel testing data, it is necessary to use complementary molecular methods designed for the detection of large genomic rearrangements. The study’s core novelty is providing compelling clinical justification for a two-tier diagnostic strategy for Lynch syndrome (LS), confirming that the high sensitivity of (NGS) for small variants must be complemented by multiplex ligation-dependent probe amplification (MLPA) for copy number variation (CNV) detection when the NGS bioinformatics pipeline is limited. This combined approach, while having a slightly higher upfront cost, offers superior long-term costs/utility by significantly reducing the rate of missed diagnoses, which prevents costly late-stage cancer treatment and facilitates life-saving cascade testing in family members. This is particularly relevant for populations where comprehensive NGS panels validated for CNV calling are not yet standard.

## Figures and Tables

**Table 1:** Relevant data for patients with pathogenic variants in MMR genes. For each patient, relevant personal and family history information are presented, as well as the results of MSI (microsatellite instability) and/or IHC (immunohistochemical) testing (if the tumor tissue was available to perform the analysis). Also, results from NGS or MLPA testing relevant to LS diagnosis are shown.

Sample Number	Age, Gender	Relevant Information from Personal History	Relevant Information from Family History	IHC/MSI Testing Results	NGS/MLPA Testing Results (Type of Genomic Change)
1	52, M	UC; CC (at 49 y.o)	Mother—EC, CC (at 60 y.o) and KC; cousins (on the mother’s side)—CC (between 45 and 50 y.o)	No data.	*MSH2* del ex1 and upstream (CNV)
2	48, F	LS in family	Mother—BC, KC, aCC with liver metastases; paternal grandmother—BC (60 y.o); father’s sister—BC (60 y.o)	No data.	*MSH6* c.741dupA (p.Arg248ThrfsTer8) (Frameshift variant)
3	45, F	Sigmoid CC (42 y.o) Adenocarcinoma of the uterus and ovaries (31 y.o)	Father—CC (67 y.o)	MLH1+, PMS2+, MSH2-, MSH6-.	*MSH2* c.1408delG (p.Val470Ter) (Nonsense variant)
4	43, F	aCC (40 y.o)	Father, two uncles, and paternal cousin—CC (all before 40 y.o); both grandmothers—BC (over 70 y.o)	MSH2+, MSH6+, MLH1-, PMS2-.	*MLH1* del ex 9–15 (CNV)
5	45, M	CC (43 y.o)	Mother—CC (39 y.o); brother—CC (27 y.o) brother’s daughter—brain tumor (21 y.o); father—SC	MSI-H.	*MLH1* c.306+1G>A (Splice donor)
6	59, F	Malignant neoplasm of the ascending colon (45 y.o)	Son—CC (30 y.o); brother 1—CC (36 y.o) brother 2—pancreatic cancer (69 y.o); mother—CC (49 y.o); mother’s sister and her daughters—CC; mother’s brother—CC (40 y.o)	No data.	*MLH1* c.199G>A (p.Gly67Arg) (Missense variant)
7	55, F	Cervical cancer (47 y.o)	Mother—cervical cancer (52 y.o); ureteral cancer (65 y.o); CC (67 y.o); five maternal uncles—CC; cousin—CC (23 y.o)	No data.	*MSH2* c.366+1G>A (Splice donor)
8	54, M	CC (52 y.o)	Mother and aunts on the maternal side—SC; cousin and uncle—CC (both in their 50s)	No data.	*MSH2* c.518T>C (p.Leu173Pro) (Missense variant)
9	31, F	CC (28 y.o.)	Positive for CC, suspected LS	MSI-H tumor MLH1+, PMS+ MSH6-, MSH2-.	*MSH2* del ex1 and upstream (CNV)
10	76, F	BC (60 y.o); Cecum cancer (68 y.o); GC (69 y.o)	Mother—plasmacytoma (85 y.o); brother—SC (65 y.o); sister—UtC (48 y.o)	No data.	*MSH6* c.2680C>T (p.Gln894Ter) (Nonsense variant)
11	41, F	BC (38 y.o)	Grandmother’s sister on the mother’s side—BC (60 y.o); father—CC (45 y.o); father’s uncle—pancreatic cancer; father’s aunt—UtC; uncle—lung cancer	No data.	*MSH6* c.3959_3962del (p.Ala1320GlufsTer6) (Frameshift variant)
12	35, M	CC (33 y.o)	Father—esophageal cancer (41 y.o); paternal grandfather—SC (age unknown); mother—adenocarcinoma of the uterus (43 y.o); mother’s sister—UtC, and later BC; maternal grandmother — UtC	No data.	*MLH1* c.67del (p.Glu23LysfsTer13) (Frameshift variant)
13	36, F	EC and ovarian cancer (33 y.o)	Uncle—lung cancer (before the age of 50); father—CLL; maternal great-grandfather—CC at a young age; mother—CC (50 y.o) mother’s sister—BC (40 y.o), UtC (53 y.o), and later bladder cancer	No data.	*MSH2* del ex 1 (CNV)
14	47, M	No malignancies.	Father—CC (62 y.o); paternal grandfather—CC (59 y.o); uncle—CC (41 y.o), and his son at the age of 32; another uncle’s son was diagnosed with CC at the age of 31. Another uncle—CC (65 y.o); paternal niece—CC (38 y.o); sister—UtC and ovarian cancer (32 y.o)	No data.	*MSH2* c.2038C>T (p.Arg680Ter) (Nonsense variant)
15	45, F	EC, UC, CC (34 y.o)	Positive family history for CC, small intestine cancer, EC, ovarian cancer, intestinal polyposis, and Hodgkin’s lymphoma.	No data.	*MLH1* del ex 9–15 (CNV)
16	72, M	CC (70 y.o)	Several family members have been diagnosed with CC, with the youngest member diagnosed at the age of 28. Several family members also had UtC and KC.	No data.	*MLH1* del ex 9–15 (CNV)
17	44, F	EC (33 y.o)	Father—CC (54 y.o); paternal grandmother—CC (43 y.o); father’s sister—ovarian cancer (45 y.o), her son was diagnosed with bladder cancer (47 y.o)	MSI-H MSH6-, MSH2-.	*MSH2* del ex 9 (CNV)

Abbreviations in alphabetical order: aCC—*adenocarcinoma of the colon*; BC—*breast cancer*; CC—*colon cancer*; CLL—*chronic lymphoblastic leukemia*; CNV—*copy number variant*; CRC—*colorectal cancer*; EC—*endometrial cancer*; F—*female*; GC—*gastric cancer*; KC—*kidney cancer*; LS—*Lynch syndrome*; MSI—*microsatellite instability*; MSI-H—*microsatellite instability—high*; IHC—*immunohistochemical testiting*; M—*male*; *MLH1—MutL Homolog 1*; MLPA—*multiplex ligation-dependent probe amplification*; MMR—*mismatch repair*; *MSH2—MutS Homolog 2*; *MSH6—MutS Homolog 6*; NGS—*next generation sequencing*; SC—*stomach cancer*; UC—*urothelial carcinoma*; UtC—*uterine cancer*; y.o—*years old*.

**Table 2:** MSI/IHC results aligning with the germline findings in five patients with a known tumor immunophenotype and proven detected germline pathogenic variant in MMR genes.

Patient Sample No. (from Table 1)	Gene and Type of Germline Pathogenic Variant (PV)	Tumor Immunophenotype (IHC/MSI)	Concordance
**3**	*MSH2* c.1408delG (Nonsense variant)	IHC: MLH1+, PMS2+, **MSH2-**, **MSH6-**	**Concordant**. MSH2 PV leads to loss of MSH2 and its binding partner, MSH6.
**4**	*MLH1* del ex 9–15 (CNV)	IHC: MSH2+, MSH6+, **MLH1-**, **PMS2-**	**Concordant**. MLH1 CNV leads to loss of MLH1 and its binding partner, PMS2.
**5**	*MLH1* c.306+1G>A (Splice donor)	MSI: **MSI-H**	**Concordant**. *MLH1* PV predicts dMMR, which is confirmed by MSI-H.
**9**	*MSH2* del ex1 and upstream (CNV)	MSI: **MSI-H**; IHC: MLH1+, PMS+, **MSH6-**, **MSH2-**	**Concordant**. MSH2 CNV leads to loss of MSH2 and MSH6.
**17**	*MSH2* del ex 9 (CNV)	MSI: **MSI-H**; IHC: **MSH6-**, **MSH2-**	**Concordant**. MSH2 CNV leads to loss of MSH2 and MSH6.

## Data Availability

Data is contained within the article or Supplementary Materials. The original contributions presented in this study are included in the article/Supplementary Materials. Further inquiries can be directed to the corresponding author.

## Supplementary Materials

The following supporting information can be downloaded at https://www.mdpi.com/article/10.3390/diagnostics15232950/s1.
